# Prognostic value of proliferation assay in the luminal, HER2-positive, and triple-negative biologic classes of breast cancer

**DOI:** 10.1186/bcr3084

**Published:** 2012-01-06

**Authors:** Mohammed A Aleskandarany, Andrew R Green, Ahmed A Benhasouna, Fabricio F Barros, Keith Neal, Jorge S Reis-Filho, Ian O Ellis, Emad A Rakha

**Affiliations:** 1 Department of Histopathology, School of Molecular Medical Sciences, Queens Medical Centre, University of Nottingham and Nottingham University Hospitals NHS Trust, Nottingham, NG7 2UH, UK; 2 Pathology Department, Faculty of Medicine, Menoufia University, Shebin El-Kom, Menoufia Governorate, Egypt; 3 Occupational Health, School of Molecular Medical Sciences, Queens Medical Centre, University of Nottingham and Nottingham University Hospitals NHS Trust, Nottingham, NG7 2UH, UK; 4 Breakthrough Breast Cancer Research Centre, Institute of Cancer Research, 237 Fulham Road, London, SW3 6JB, UK

## Abstract

**Introduction:**

Although the prognostic significance of proliferation in early invasive breast cancer has been recognized for a long time, recent gene-expression profiling studies have reemphasized its biologic and prognostic value and the potential application of its assessment in routine practice, particularly to define prognostic subgroups of luminal/hormone receptor-positive (HR^+^) tumors. This study aimed to assess the prognostic value of a proliferation assay by using Ki-67 immunohistochemistry as compared with mitotic count scores.

**Method:**

Proliferation was assessed by using Ki-67 labeling index (Ki-67LI) and mitotic scores in a large (*n *= 1,550) and well-characterized series of clinically annotated primary operable invasive breast cancer with long-term follow-up. Tumors were phenotyped based on their IHC profiles into luminal/HR^+^, HER2^+^, and triple-negative (TN) classes. We used a split-sample development and validation approach to determine the optimal Ki-67LI cut-offs.

**Results:**

The optimal cut-points of Ki-67LI were 10% and 50% for the luminal class. Both Ki7LI and MS were able to split luminal tumors into subgroups with significantly variable outcomes, independent of other variables. Neither mitotic count scores nor Ki-67LI was associated with outcome in the HER2^+ ^or the TN classes.

**Conclusions:**

Assessment of proliferation by using Ki-67LI and MS can distinguish subgroups of patients within luminal/hormone receptor-positive breast cancer significantly different in clinical outcomes. Overall, both Ki-67 LI and mitotic-count scores showed comparable results. The method described could provide a cost-effective method for prognostic subclassification of luminal/hormone receptor-positive breast cancer in routine clinical practice.

## Introduction

Tumor proliferative activity represents one of the most thoroughly investigated cellular functions in breast cancer (BC) for its association with tumor behavior [1]. Assessment of proliferation rates has been shown to provide useful information on prognosis and aggressiveness of individual cancers and can potentially be used to guide treatment protocols in clinical practice. Recently, a meta-analysis of publicly available breast cancer gene-expression signatures has identified proliferation as the key biologic driver in all nine prognostic signatures included in the study [2]. It was also demonstrated that the assignment of basal-like and ERBB2^+ ^(HER2^+^) breast cancers to the poor-prognosis groups by first-generation gene signatures is determined mainly by high expressions of proliferation-related genes [2,3]. Moreover, it has been reported that expression of genes involved in cell proliferation is the most heavily weighted component in calculating the recurrence score [4] and is the basis of genomic grading [5,6].

Various techniques have been developed to quantify proliferation rates, including, mitotic-count estimates, measurement of DNA synthesis, and flow cytometry [1,7-9]. Newer techniques include detection of antigens closely associated with proliferation by using immunohistochemistry (IHC). In theory, the latter methods are quicker, cheaper, and easier to use than flow cytometry and autoradiography and are more reproducible than is mitotic figure counting [7]. Although most studies of different proliferation assays displayed significant agreement in outcome predictions for individual patients, no consensus exists on the best proliferation assay [10]. Mitotic-count scores (MSs) are used in routine BC histologic grading, and the prognostic significance is well established [11,12]. Immunohistochemical expression of Ki-67 is now widely used as an IHC measure of proliferation [13-15]. Although Ki-67 is not yet incorporated into routine pathology practice, its use in proliferation assays in translational studies has yielded promising results [16-18]. Several studies have sub-classified luminal tumors (that is, estrogen receptor (ER)-positive, HER2 negative) based on differential expression of proliferation-associated genes "proliferation signature," gene-expression profiling (GEP), or by Ki-67 in IHC studies [16,17,19]. However, some technical and validation issues are to be addressed before Ki-67 is used in BC routine practice. It remains to be determined whether Ki-67 IHC outperforms mitotic counting as a predictor of outcome. Moreover, evidence of the prognostic value of using MS in an algorithm for defining molecular BC classes is yet to be elucidated.

Consequently, this study was conducted on a large, well-characterized series of early (stage I through III) invasive BC with long-term follow-up to study the prognostic value of MS and Ki-67LI, as methods of proliferation assays, in different BC molecular classes. We used a split-sample development and validation approach to determine the optimal Ki-67LI cut-offs and demonstrated its prognostic relevance in the validation cohort in luminal/HR^+^, HER2^+^, and triple-negative tumors.

## Materials and methods

### Patients and tumors

This study was based on a well-characterized cohort of early-stage (I-III) primary operable invasive BC from patients aged 70 years or younger, enrolled into the Nottingham Tenovus Primary Breast Carcinoma Series between 1990 and 1998 (*n *= 1,550), and managed in accordance with a uniform protocol. Patients' clinical history, tumor characteristics, and information on therapy and outcomes are prospectively maintained. Outcome data include survival status, survival time, cause of death, development, and time to locoregional recurrence and distant metastasis (DM). Breast Cancer Specific Survival (BCSS) is defined as the time (in months) from the date of primary surgery to the date of breast cancer-related death. DMFS is defined as the time (in months) from the date of primary surgery to the appearance of distant metastasis.

Patients had a median age of 54 years (range, 18 to 70 years) with a median overall survival of 123 months (range, 4 to 234 months) and a median time of event-free survival of 110 months (range, 3 to 226 months). Distant recurrence occurred in 443 (30.4%) cases; 392 (26.9%) patients died of BC; and 747 (58.1%) patients were alive at the end of follow-up.

### Assessment of mitotic counts

Mitotic figures were counted by one of three pathologists with extensive experience in BC histopathology from Nottingham City Hospital, Nottingham, UK (Ian Ellis, Sarah Pinder, and Christopher Elston), in 10 high-power fields, as part of routine BC histologic grading [20]. Scores of 1 to 3 were used, with cut-off points determined based on association with outcome (MS 1, ≤ 9 mitoses/10 hpf; MS 2, 10 to 19 mitoses/10 hpf; and MS 3, ≥ 20 mitoses/10 hpf) by using a 0.59-mm microscopic field diameter [21].

### Ki-67 immunohistochemistry

Formalin-fixed paraffin tissue sections (FFSs, 4 μm) mounted on Superfrost slides (Surgipath) were immunohistochemically stained, by using the standard streptavidin-biotin complex method, as previously described [22]. Microwave-assisted heat-induced retrieval method for antigen epitopes was performed in citrate buffer, at pH 6.0 for 20 minutes. Endogenous peroxidase activity was blocked by incubation in a 0.3% hydrogen peroxide in methanol buffer for 10 minutes. Nonspecific binding of primary antibody was blocked by using normal swine serum (NSS, in Tris-buffered saline (TBS) (1:5), 100 μl/slide) for 10 minutes of incubation. Primary mouse monoclonal anti-Ki-67 antibody (MIB1 clone, product M7240; Dako, Glostrup, Denmark), diluted 1:100 (optimum working dilution) in NSS/TBS, was applied to each slide and incubated for 60 minutes at room temperature. Slides were then rinsed in TBS before staining with a streptavidin-biotin three-stage technique, with the Dako Strept ABC complex/HRP Duet kit (Dako, K492) according to manufacturer's guidelines. For reaction visualization, 3-3 diaminobenzidine tetrahydrochloride (Dako liquid DAB Plus, K3468) was used as chromogen. The sections were counterstained with Mayer hematoxylin (Dako, AR106). Human tonsil sections were used as positive control, whereas negative control was performed by replacing the primary antibody by TBS. These controls were included in each staining run. Additionally, to assess the optimal number of tumor blocks from an individual case sufficient to report on Ki-67LI, four FFSs cut from four different paraffin blocks, representative of 25 invasive BC cases, also were stained.

#### Ki67 scoring

Immunostaining was quantitatively evaluated by using light microscopy, in which the entire section was scanned at low-power magnification (×100) to determine areas with the highest numbers of positive nuclei (hot spot) within the invasive component [22]. These were usually found at the periphery of tumors and were easier to identify than the mitotic figure hot spots. Ki-67 labeling index (Ki-67LI) was expressed as the percentage of MIB1-positive cells among a total number of 1,000 malignant cells at high-power magnification (×400). A randomly chosen subset of cases (*n *= 200) was rescored by the same observer (MA), and an intraobserver reproducibility test was performed (kappa value, 0.85).

### Definition of molecular classes

Data on a wide range of biomarkers of known clinical and biologic relevance to BC were available, including hormone receptors [HR, ER, and progesterone receptor (PR)], epidermal growth factor-receptor family members (HER1 (EGFR), and HER2) and basal cytokeratins (CKs) (CK5/6 and CK14) [23,24]. In this study, HER2 was assessed by using IHC and dual-color chromogenic *in situ *hybridization (CISH), as previously published [25]. Moreover, data on a subset of 128 frozen BC tissues that were profiled by using Illumina WG-6 BeadChips, as previously described [26], was available. These expression data are available at the EBI website http://www.ebi.ac.uk/miamexpress/ with E-TABM-576 accession number.

Molecular classes were defined as luminal (HR^+^: ER^+ ^and/or PR^+^]), HER2^+ ^(HER2^+ ^regardless of the expression of other markers), basal-like [BLBC] (ER^-^, PR^-^, HER2^-^, and positive for CK5/6, and/or CK14 and/or EGFR), and triple-negative nonbasal BC (TNnon-B; all these markers negative) [23,27].

To assess the optimal Ki-67 LI cut-off in different molecular classes and to avoid data overfitting, each molecular class was randomly split into two subsets by using SPSS random sampling; one third of cases were used as a training set, and the remaining two thirds used as a validation (test) set. No differences between training and validation sets were identified in any of the molecular classes (Table 1).

**Table 1:** Clinicopathologic features of the whole

Characteristics	Whole series	Luminal class			HER2^+ ^class			Triple-negative class		
		
		Training No (%)	Validation No (%)	*P*	Training No (%)	Validation No (%)	*P*	Training No (%)	Validation No (%)	*P*
**Age**										
Median (Range)	54 (18-70)	55 (18-70)	55 (28-70)	0.690	52 (29-69)	52 (27-70)	0.657	49.5 (29-70)	50 (27-70)	0.885
**Menopausal status**										
Premenopausal	571 (39.2)	116 (36.7)	232 (35.6)	0.887	31 (47.7)	58 (45.3)		44 (54.8)	90 (56.3)	0.892
Postmenopausal	886 (60.8)	200 (63.3)	419 (64.4)		34 (52.3)	70 (57.7)	0.762	36 (45.1)	70 (34.8)	
**Tumor grade**										
Grade I	255 (17.5)	80 (25.3)	151 (23.2)		2 (3.1)	5 (3.9)		3 (3.6)	1 (0.6)	0.892
Grade II	479 (32.9)	141 (43.9)	282 (43.3)	0.371	8 (12.3)	25 (19.5)	0.417	7 (8.3)	8 (5.0)	
Grade III	723 (48.6)	95 (29.6)	218 (33.5)		55 (84.6)	98 (76.6)		70 (88.1)	151 (94.4)	
**Tumor size**										
≤ 2 cm	899 (61.8)	215 (68.1)	433 (66.5)	0.561	40 (61.5)	77 (60.2)	0.877	42 (52.5)	71 (44.4)	0.180
> 2 cm	556 (38.2)	101 (31.9)	218 (33.5)		25 (38.5)	51 (39.8)		38 (47.5)	89 (55.6)	
**Tumor type**										
Ductal, no special type	853 (58.5)	159 (50.3)	331 (50.8)	0.874	59 (90.8)	112 (88.2)	0.807	70 (87.5)	136 (86.1)	1.00
Other histologic types	604 (41.5)	157 (49.7)	320 (49.2)		6 (9.2)	15 (11.8)		12 (12.5)	22 (13.9)	
**NPI**^a ^										
Good	457 (31.4)	141 (44.6)	275 (42.2)		4 (6.5)	16 (12.5)		7 (8.6)	13 (8.1)	
Moderate	767 (52.6)	145 (45.9)	295 (45.3)	0.312	47 (72.3)	78 (60.9)	0.226	55 (68.9)	109 (68.1)	0.417
Poor	233 (16.0)	30 (9.5)	81 (12.4)		14 (21.5)	34 (26.6)		18 (22.5)	38 (23.8)	
Mean (range)	3.4 (2.04-5)	3.42 (2.05-5)	3.43 (2.06-5)		4.7 (2.6-5)	4.7 (2.6-5)		4.51 (2.05-5)	4.52 (2.05-5)	
**Recurrence**										
Local: No	1,249 (89.0)	283 (89.6)	588 (90.3)		55 (84.6)	108 (84.4)		70 (87.5)	136 (85.0)	0.564
Yes	160 (11.0)	33 (10.4)	63 (9.7)	0.819	10 (15.4)	20 (15.6)	1.000	10 (12.5)	24 (15.0)	
Regional: No	1,280 (91.0)	291 (92.1)	594 (91.2)		58 (89.2)	112 (87.5)		70 (87.5)	147 (91.9)	0.259
Yes	129 (9.0)	25 (7.9)	57 (8.8)	0.713	7 (10.8)	16 (12.5)	0.817	10 (12.5)	13 (8.1)	
Distant: No	1,014 (69.6)	239 (75.6)	473 (72.7)	0.278	34 (52.3)	71 (55.5)		50 (62.5)	107 (66.9)	0.573
Yes	443 (30.4)	77 (24.4)	178 (27.3)		31 (47.7)	57 (44.5)	0.760	30 (37.5)	53 (33.1)	

^a^Series (*n *= 1,457), training sets, and validation sets of luminal, HER2^+ ^(*n *= 193), and TN (*n *= 244) molecular BC classes Nottingham Prognostic Index. Good Prognostic Group, ≤ 3.4; Moderate Prognostic Group, 3.41 to 5.4, Poor Prognostic Group, > 5.4

### Statistical analysis

Statistical analysis was performed by using SPSS 15.0 statistical package (SPSS Inc., Chicago, IL, USA). X-tile software (version 3.6.1, 2003-2005, Yale University, New Haven, CT, USA) [28] was used to de-termine the optimal Ki-67LI cutoff point(s) in different molecular subtypes. Correlations between Ki-67LI and MS and other variables were studied with a χ^2 ^test, Fisher Exact test, and Mann-Whitney *U *test. Survival curves for BCSS and DMFS were drawn by using the Kaplan-Meier estimates, and significance was assessed by using log-rank tests. Multivariate analyses of BCSS and DMFS were conducted by using Cox proportional hazard regression models. A two-tailed *P *value < 0.05 was considered significant.

This study was approved by the Nottingham Research Ethics Committee 2 under the title "Development of a molecular genetic classification of breast cancer." All patients who participated in this study gave their written informed consent at the time of their donation.

## Results

In the current study, informative results for Ki-67LI, MS, and follow-up data were available for 1,457 cases (94% of the whole series). To assess the optimal number of FFSs sufficient to report Ki-67LI reliably, four FFSs were cut from four different paraffin blocks representative of 25 invasive BC cases. These showed high levels of concordance between sections when Ki-67LI was analyzed as continuous variable (*P *< 0.0001; Table 2) and when categorized by using different cut-off points (kappa value, 0.834; 95% CI, 0.76 to 0.92), indicating that one FFS is sufficient for reliable Ki-67LI assessment.

**Table 2:** Results of multivariate analysis of variance (MANOVA) test for Ki-67LI assessed on FFS from 25 invasive BC cases

Factor	Mean Ki-67LI	Standard error	95% confidence interval		MANOVA	*P *value
			Lower bound	Upper bound		
Section 1	71.280	4.701	62.376	81.184	14.960	< 0.0001
Section 2	74.800	4.034	66.475	83.125		
Section 3	71.800	4.709	62.082	81.518		
Section 4	74.160	4.054	65.793	82.527		

Presented as one section per block; four sections per case.

**Figure 1:**
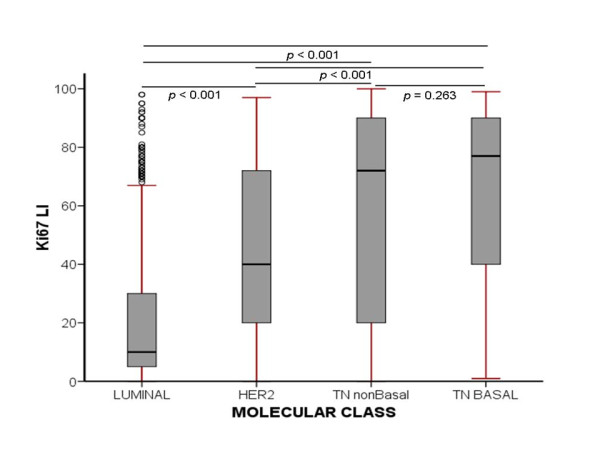
**Box plot of Ki-67LI in different BC molecular classes, and their corresponding levels of significance in *post hoc *analysis**.

**Figure 2:**
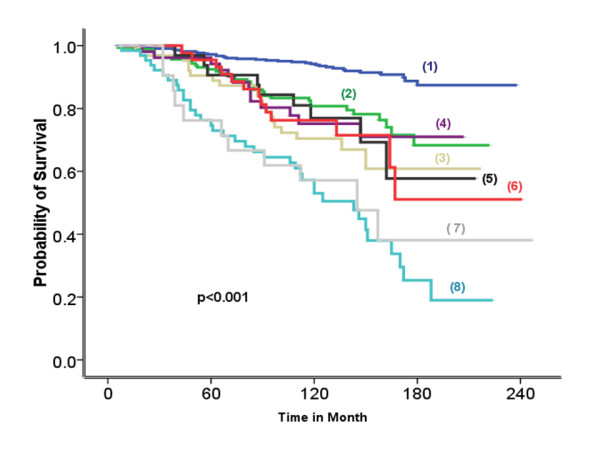
**Association between BCSS and Ki-67LI expressed in 10% increments (10% each.) However, tumors showing 50% to 69% and 80% to 100% Ki-67LI were considered as two groups, as the number in each 10% subgroup was small)**. Labels 1 through 8 represent patients' subsets based on tumor Ki-67LI, where 1 is 0 to 9%; 2 is 10% to 19%; 3 is 20% to 29%; 4 is 30% to 39%; 5 is 40% to 49%; 6 is 50% to 69%; 7 is 70% to 79%; and 8 is 80% to 100%.

Significant association was observed between Ki-67LI and MKI67 gene transcript, as defined by microarray-based gene-expression profiling (*r^2 ^*= 0.24; *P *< 0.0001), and between Ki-67LI and MS (*P *< 0.0001). High Ki-67LI and MS were associated with premenopausal status, larger tumor size, definite vascular invasion, and lymph node involvement (*P *< 0.0001). Significant differences appeared between BC molecular classes regarding their Ki-67LI (ANOVA, *F *= 167; *P *< 0.0001) (Figure 1). Bonferroni *post hoc *testing of Ki-67LI revealed significant differences between luminal (lowest Ki-67LI) and HER2^+ ^(intermediate Ki-67LI) and between HER2^+ ^and triple-negative (highest Ki-67LI) classes. However, the difference between BLBC and TNnon-B was not significant (*P = *0.263). Similar results were found when MS was used to define proliferation. When the prognostic results of Ki-67LI were expressed as a continuous variable in the class of luminal ER^+^/HER2^- ^tumors, this showed association with outcome (χ^2 ^= 80; *P *< 0.0001; HR = 1.1; 95% CI, 1.0 to 1.2). Moreover, Ki-67LI cut-offs at 10% increments within the same class showed statistically significant differences between the resulting patients' subsets (LR = 142.64; *P *< 0.0001; HR = 1.3; 95% CI = 1.2 to 1.3; Figure 2).

### Determination of the optimal Ki-67LI cut-offs

**Figure 3:**
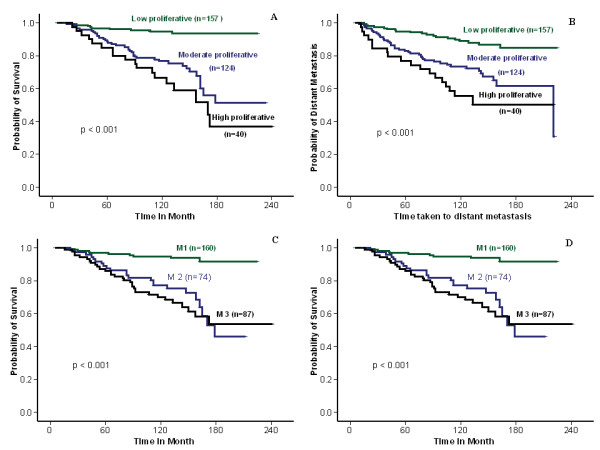
**Kaplan-Meier survival plot for luminal BC training set using Ki-67LI and MS**. **(a) **Breast cancer-specific survival (BCSS). **(b) **Metastasis-free survival at 10% and 50% Ki-67LI cut-off point. **(c, d) **BCSS and DMFS for mitosis-frequency scores.

In the training sets, the prognostic significance of previously defined Ki-67LI cut-off points including 10%, 13%, 17% (median), 20%, 25%, 30%, 35% [16,27,29,30] were assessed (Table 3). This shows that 10% cut-off had the highest hazard ratios in the luminal class, but none of these cut-offs was statistically significant in HER2^+ ^or TN classes. The optimal Ki-67LI cut-offs within each molecular class was then assessed by using X-tile software analysis [28]. In the luminal class, this showed that 10% is the optimal cut-off separating low from moderate/high proliferative subgroups. In addition, it showed that 50% cut-off value can split the latter subgroup into two prognostically different subclasses (moderate (10% to 50%) and high proliferative subgroups (> 50%)) with a reasonable number of cases within each subclass (Figure 3). In HER2^+ ^and TN classes, only one cut-off could be identified (75% and 70%, respectively). Table 4 displays MS, training and validation sets, and Ki-67LI cut-off points in BC molecular classes.

**Table 3:** Hazard ratios for different Ki-67LI cut-off points for BCSS and DMFS in luminal and HER^+ ^training sets

Cut-off point	Luminal BC				HER2^+ ^BC			
	
	BCSS		DMFS		BCSS		DMFS	
	*P *	HR (95% CI)	*P *	HR (95% CI)	*P *	HR (95% CI)	*P *	HR (95% CI)
10%	< 0.001	4.289 (3.043-6.045)	< 0.001	2.951 (2.233-3.901)	0.439	1.314 (0.658-2.625)	0.401	1.334 (0.674-2.682)
13%		3.484 (2.580-4.704)		2.595 (2.006-3.356)	0.647	0.883 (0.519-1.503)	0.758	0.920 (0.541-1.536)
17%		3.387 (2.547-4.506)		2.468 (1.923-3.166)	0.318	0.780 (0.480-1.270)	0.368	0.800 (0.493-1.299)
20%		3.555 (2.683-4.712)		2.578 (2.012-3.304)	0.367	0.805 (0.502-1.291)	0.457	0.837 (0.523-1.339)
25%		3.091 (2.348-4.069)		2.458 (1.918-3.150)	0.355	0.810 (0.519-1.265)	0.483	0.854 (0.549-1.328)
30%		3.004 (2.283-3.954)		2.415 (1.880-3.101)	0.717	0.923 (0.596-1.427)	0.824	0.952 (0.617-1.468)
35%		3.182 (2.413-4.195)		2.648 (2.055-3.413)	0.698	1.089 (0.708-1.675)	0.723	1.080 (0.706-1.652)
10% and 50%:					0.191		0.334	
Moderate vs. low		3.473 (2.421-4.983)		2.483 (1.847-3.336)	0.747	1.126 (0.547-2.319)	0.611	1.205 (0.588-2.469)
High vs. low		7.648 (5.116-11.43)		5.021 (3.533-7.134)	0.188	1.638 (0.786-3.414)	0.220	1.586 (0.759-3.313)

BCSS, Breast cancer-specific survival; DMFS, distant metastasis-free survival; MS, mitosis score.

**Table 4:** Mitotic scores (MSs), training and validation sets, and Ki-67LI descriptive measures in BC molecular classes

	Luminal number (%)		HER2^+ ^number (%)	TN number (%)
**Total^a ^**	967 (69.9)		193 (13.6)	240 (16.5)
**Mitosis scores**				
1	318 (48.8)		16 (12.5)	4 (2.5)
2	143 (22)		29 (22.7)	8 (5)
3	190 (29.2)		83 (64.8)	148 (92.5)
**Training set^b^**	316 (33)		65 (33)	80 (33%)
**Validation set^b^**	651 (67)		128 (67)	160 (67)
**Ki-67LI in the validation sets**				
Ki-67 mean (SD)	22 (24.6)		41.5 (27.4)	64.5 (32)
Ki-67LI median (range)	11 (0-98)		39.5 (0-97)	77 (1-98)
Ki-67LI Cut-off	10	50	70	-

^**a**^This percentage is relative to the whole patients' series (that is, 1,457 cases). ^**b**^This percentage is relative to number of cases within the same class.

Using 10% and 50% Ki-67LI cut offs divided the luminal class into low (46.9%), moderate (41.6%), and high (11.5%) proliferation subgroups, respectively.

Ki-67LI cut-offs were generated by using X-tile software in the training sets.

### Proliferation in luminal class

**Figure 4:**
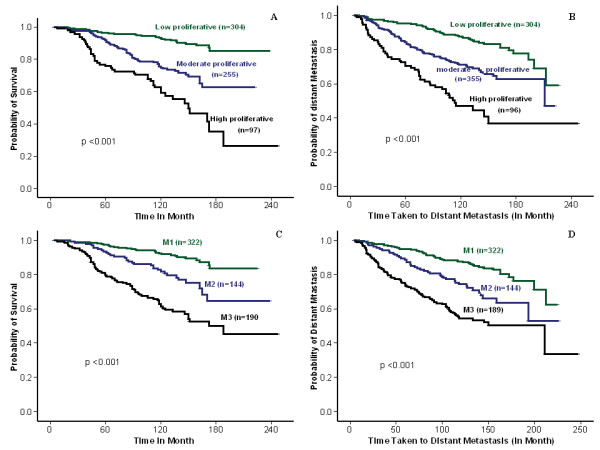
**Kaplan-Meier survival plot for luminal BC validation set using Ki-67LI and MS**. **(a) **Breast cancer-specific survival (BCSS) and **(b) **metastasis-free survival at 10% and 50% Ki-67LI cut-off point. **(c, d) **BCSS and DMFS for mitosis frequency scores (by using the validation set only).

Univariate survival analysis of the validation set revealed significant association between MS (1, 2, and 3) and patients' outcomes, including BCSS (*P *< 0.001, HR = 2.3; 95% CI = 1.9 to 2.8), and DMFS (*P *< 0.001; HR = 1.9; 95% CI, 1.6 to 2.3). Low, moderate, and high proliferative subgroups of luminal class validation set displayed significant differences in BCSS (LR = 76.75; *P *< 0.001; HR = 2.5; 95% CI, 2.0 to 3.2) and DMFS (LR = 90.87; *P *< 0.001; HR = 2.2; 95% CI, 1.8 to 2.7) (Figure 4). Multivariate analysis showed that both MS and Ki-67LI were independent prognostic factors (*P *< 0.001). However, the HR for Ki-67LI was slightly higher than that of MS, although the CIs of the high-proliferation subgroups overlapped. As Ki-67LI and MS are highly correlated, multivariate analysis including MS, tumor grade, nodal stage, and tumor size was repeated after the addition of Ki-67LI. This showed that Ki-67LI has a higher HR than that of MS for both BCSS and DMFS; Table 5.

**Table 5:** Cox proportional hazards analysis for predictors of BCSS and DMFS in luminal BC valida-tion set: effect of Ki-67LI, MS, tumor grade, nodal stage, tumor size, and adjuvant therapy

Variables	Luminal BC			
	
	BCSS		DMFS	
	*P *value	HR (95% CI)	*P *value	HR (95% CI)
Ki-67LI	0.019		0.031	
Low				
Moderate	0.028	1.945 (1.076-3.513)	0.258	1.319 (0.816-2.132)
High	0.005	2.679 (1.347-5.330)	0.013	2.097 (1.171-3.754)
Mitosis scores	0.028		0.015	
1				
2	0.043	1.403 (0.723-2.723)	0.055	1.228 (0.801-2.002)
3	0.033	2.096 (0.938-4.687)	0.018	1.854 (0.919-3.741)
Tumor grade	0.022		0.024	
1				
2	0.016	1.379 (0.647-2.938)	0.041	1.604 (0.878-2.930)
3	0.014	1.595 (0.621-4.096)	0.017	1.566 (0.909-3.459)
Nodal stage	0.001		0.001	
Node negative				
1 to 3 positive LN	0.001	1.969 (1.331-2.913)	0.003	1.839 (0.297-2.607)
≥ 4 positive LN	0.002	3.498 (2.069-5.915)	0.005	3.330 (2.070-5.358)
Tumor size	0.221	1.262 (0.869-0.834)	0.019	1.492(1.067-2.087)
Endocrine therapy (No/Yes)	0.378	0.553 (0.324-0.499)	0.961	0.998 (0.904-1.101)
Chemotherapy (No/Yes)	0.030	0.768 (0.451-0.309)	0.061	0.636 (0.397-1.021)

BCSS, Breast cancer-specific survival; DMFS, distant metastasis-free survival; MS, mitosis score.

Interestingly, the HRs for both BCSS and DMFS were highest for Ki-67LI, followed by histologic grade, HER2 status, and then by MS, whereas the lowest HR was achieved by PR status; Table 6.

**Table 6:** Cox proportional hazards analysis for predictors of BCSS in luminal BC validation set: effect of Ki-67LI, MS, tumor grade, HER2 status, PR status, and adjuvant therapy

Variables	Luminal BC			
	
	BCSS		DMFS	
	*P *value	HR (95% CI)	*P *value	HR (95% CI)
Ki-67LI	0.006		0.003	
Low				
Moderate	0.020	2.016 (1.118-3.636)	0.211	1.359 (0.840-2.198)
High	0.002	3.002 (1.522-5.923)	0.002	2.504 (1.410-4.448)
Tumor grade	0.031		0.021	
1				
2	0.018	1.696 (0.783-3.672)	0.055	1.817 (0.987-3.346)
3	0.023	1.736 (0.634-4.753)	0.011	1.556 (0.986-3.659)
HER2 status	0.021	1.528 (1.022-2.385)	0.042	1.263 (0.982-2.746)
Mitosis scores	0.018		0.044	
1				
2	0.043	1.267 (0.651-2.466)	0.013	1.292 (0.742-2.257)
3	0.086	2.113 (0.899-4.967)	0.017	1.877 (0.881-3.999)
PR status	0.030	0.635 (0.422-0.956)	0.064	0.701 (0.481-1.021)
Endocrine therapy (No/Yes)	0.886	0.992 (0.890-1.106)	0.314	1.049 (0.955-1.152)
Chemotherapy (No/Yes)	0.332	0.768 (0.451-0.309)	0.640	0.893 (0.557-1.432)

**Figure 5:**
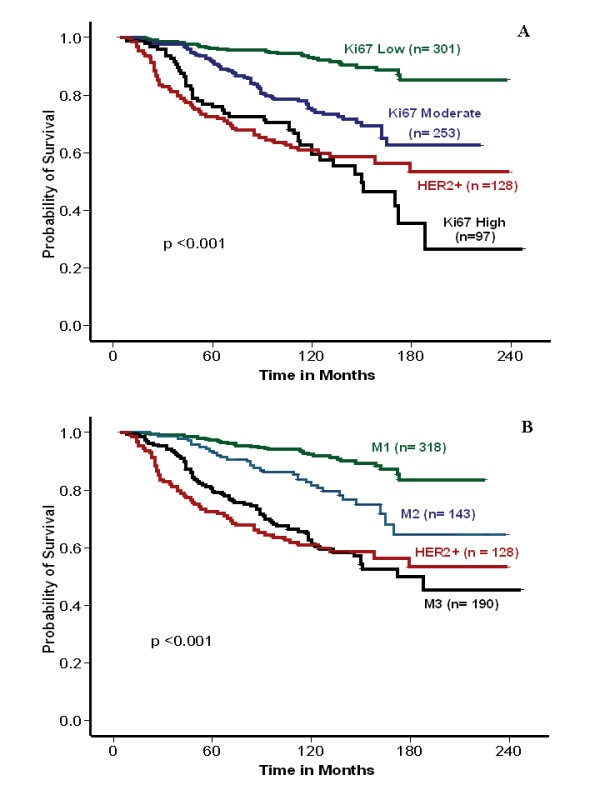
**Kaplan-Meier survival plot of luminal tumors showing association between HER2 status and proliferation**. (Ki-67LI, **a**, and MS, **b**) and BCSS (LR = 88; *P *< 0.0001; HR, 1.683; 95% CI, 1.492 to 1.898, and LR, 90.239; *P *< 0.0001; HR, 1.749; 95% CI, 1. 543 to 1.982, respectively; validation set only). Number of patients at risk is shown above the curves.

To assess the difference between Ki-67 and HER2 status in HR^+ ^cancers, luminal tumors were further classified after inclusion of HR^+^/HER2^+ ^tumors [31,32] into luminal 1 and 2, with the former being HR^+ ^and HER2^- ^(luminal), and the later being HR^+ ^and HER2^+^. In luminal 1 subclass, both Ki-67LI and MS were associated with BCSS and DMFS, independent of other variables. However, no associations were found between either MS or Ki-67LI with BCSS or DMFS in luminal 2 subclass by using either 10% and 50% or 75% Ki-67LI cut-offs. When HER2 status and proliferation were considered within HR^+ ^tumors, distinct subclasses with prognostic significance were identified (Figure 5). Ki-67LI and MS classified HR^+ ^tumors into low (excellent outcome) and moderate proliferative activity (moderately poor outcome), whereas the high-proliferative group was associated with the worst outcome that was not different from HER2^+ ^tumors (Table 7). Similar results were obtained after adjustment for chemotherapy. Multivariate analysis including size, nodal stage, VI, systemic therapy, Ki-67LI, and MS showed that Ki-67LI HR (1.6; 95% CI, 1.2 to 2.1) is similar to that of stage (1.8; 95% CI, 1.4 to 2.2) and HER2 (1.7; 95% CI, 1.3 to 2.6), and higher than that of MS (1.4; 95% CI, 1.1 to 1.8).

**Table 7:** Ten-year survival rates of hormone receptors positive (ER^+ ^and/or PR^+^)/luminal class (including HER2^+ ^cases) based on proliferation assay (Ki-67LI and mitotic scores) and HER2 status

Variable	BCSS			DMFS		
	Number exposed to risk	Number of events	Proportion surviving	Number exposed to risk	Number of events	Proportion surviving
Ki-67LI						
Low	295	28	90.5%	295	46	84.4%
Moderate	250	68	72.8%	249	77	69.7%
High	95	43	54.7%	94	50	46.8%
Mitotic score						
1	324	39	88%	324	58	82%
2	156	36	77%	155	46	70.3%
3	226	93	58.8%	225	101	55%
HER2 status						
Negative	622	136	78.1%	620	170	72.5%
Positive	66	29	56%	66	32	51.5%

BCSS, Breast cancer-specific survival; DMFS, distant metastasis-free survival.

In addition, analysis of 70 luminal tumors defined by global gene-expression profiling [26] showed significant association between Ki-67LI and BCSS (*P *= 0.011; HR, 2.2; 95% CI, 1.2 to 4.1) and DMFS (*P *= 0.016; HR, 2.1; 95% CI, 1.1 to 3.7), and between MS and BCSS (*P *= 0.024; HR, 1.8; 95% CI, 1.1 to 3.1) and DMFS (*P *= 0.015; HR, 1.9; 95% CI, 1.1 to 3.2).

### Proliferation in HER2-positive breast cancer

Kaplan-Meier plots showed no association between MS and either BCSS (*P = *0.611; HR, 1.1; 95% CI, 0.7 to 1.5) or DMFS (*P = *0.617; HR, 1.0; 95% CI, 0.7 to 1.5) in HER2^+ ^tumors. Combining MS1 and 2 and repeated testing against MS3 yielded the same results. Although 75% Ki-67LI cut-off split the HER2^+ ^training set into low/moderate (< 75%; 80% of cases) and high-proliferative (≥ 75%; 20% of cases) subclasses with difference in outcomes, survival analysis within the HER2^+ ^validation set showed no significant difference between these two subgroups for either BCSS (*P *= 0.445) or DMFS (*P *= 0.784).

### Proliferation in triple-negative breast cancers

Univariate survival analysis revealed no association between MS and either BCSS (*P = *0.317) or DMFS (*P = *0.590) in TN cancers. Similarly, no difference was found when MS1 and 2 were combined and tested against MS3. In addition, no association was found between Ki-67LI and BCSS or DMFS by using the X-tile generated cut-point in the TN BC training set (that is, 70%; *P *= 0.174). When TN tumors were subclassified into BLBC and TNnon-B BC, Kaplan-Meier plots showed no significant association between MS and Ki-67I and either BCSS or DMFS (*P *> 0.05).

## Discussion

Although the prognostic significance of proliferation in BC has been documented and validated in several independent studies, recent global gene-expression profiling studies have reemphasised its biologic and prognostic importance. Several studies have identified the proliferation signature as a key element in the molecular classification of BC and in the composition of different prognostic and predictive gene signatures [2]. Therefore, some recent studies have raised the issue of using an immunohistochemical marker for assessment of proliferation (for example, Ki-67) to be used in combination with other IHC surrogate panels used for BC molecular classification: ER, PR, and HER2 [16,17]. These studies have demonstrated that Ki-67LI is associated with outcome and, when used in combination with other markers mentioned previously, provides valuable prognostic information and can subclassify luminal (HR^+ ^tumors) into prognostically distinct subclasses. Therefore, in the current study, we used a well-characterized series of operable invasive BC to assess the prognostic significance of Ki-67LI with regard to BC molecular classes compared with that provided by the routinely assessed MS and addressed some technical issues for use in routine practice in assessing tumor proliferation.

The distributions of Ki-67LI values in this series (mean and median) were consistent with those found in previous studies [29,33], and have a significant correlation with MKI67 gene transcript and MS. Consis-tent with the results of GEP studies [2], the majority of HER2^+ ^and TN tumors showed high proliferative activity in terms of high Ki-67LI and high MS, whereas up to half of luminal BCs were of low MS and showed low Ki-67LI.

In luminal BC, proliferation assays using MS and Ki-67LI identified subdivisions with statistically different patient outcomes. In this group, both MS and Ki-67LI retained their significant and independent association with outcome and identified three subclasses; one (low-proliferative group) associated with excellent prognosis where adjuvant chemotherapy is unlikely to provide benefit and could potentially be withheld, and one class (high-proliferative group) with a worse prognosis akin to HER2^+ ^tumors trastuzumab (Herceptin)-naïve patients) that may be an appropriate subclass likely to benefit from chemotherapy. These findings confirm the prognostic relevance of routinely assessed MS in the luminal class, as previously reported in BC [1,7,8]. It is, however, important to recognize that our results relating to MS are based on optimized and standardized methods for tissue handling, fixation, and preparation [21]. Suboptimal tissue fixation has been demonstrated to affect adversely the ability to assess mitotic frequency, resulting in a systematic downgrading of cases [34-36]. Critical evaluation of these issues with recommendations for good practice has been provided by professional organizations. Significant improvements in the consistency of assessment of mitotic counts and hence histologic grading have been observed on a national basis in the United Kingdom through publication of guidelines with linked educational activity and associated external quality assurance [34]. However, it is worth mentioning that the prognostic information obtained by Ki-67LI in luminal tumors is equivalent to or even greater (in terms of HR) than that provided by MS alone and than that provided by histologic grade or HER2 or PR status.

Different authorities have highlighted the prognostic significance of Ki-67LI in luminal BC, with emphasis on its ability to stratify patients into low-risk and high-risk populations. Although, in these studies, luminal BCs were subdivided into distinct subgroups, the optimal cut-off point remains nonstandardized, with most studies using a single, usually the median, cut-off point; dividing these tumors into two subgroups [16]. In this study, two cut-offs for Ki-67LI (10% and 50%) were identified as derived from associations with patient outcome in luminal tumors, which is the largest class of BC; three subdivisions with significantly different outcomes with adequate numbers of cases within each subgroup. In this study, the proliferative activity assessed by using Ki-67LI was compared with mitotic counts that were scored in routine practice by using full-face sections (one to four sections per tumor). Ki-67 LI was assessed by using full-face sections to avoid missing the hot spot and to provide data that can be used in routine practice rather than TMA, which is currently a research tool. Although some research studies of Ki-67LI have reported correlation between Ki-67LI assessed on TMA or needle-core biopsy [37] and that assessed on full-face sections, these correlations are not absolute, and discrepant cases exist. The results of the current study support other studies that demonstrated that Ki-67LI should be assessed in the most active areas (hot spots), a method that has also been used and validated in the assessment of mitotic counts [21].

The majority of HER2^+ ^and TN tumors are known to be poorly differentiated and highly proliferative [2]. No association between MS and outcome could be identified in either HER2^+ ^or TN classes. None of the previously reported Ki-67LI cut-off points was able to stratify HER2^+ ^or TN tumors into clinically relevant subclasses. Although the high Ki-67LI cut-off generated in HER2^+ ^and TN training sets appeared to stratify these tumors into proliferative subgroups based on outcome, no association with survival was identified in the validation sets. These findings could be explained by the small number of cases in these molecular classes after splitting them into training and validation sets or by their high proliferation rate, which limits the ability of a proliferation marker to identify clinically distinct subclasses.

The number of sections from each case required to assess Ki-67LI reliably in a clinical laboratory setting also was addressed. The results obtained from each of four sections assessed per tumor showed a high level of concordance, indicating that using a single FFS/case appears appropriate and representative.

It is important, however, to mention that in the current study, outcome was assessed in a context in which the treatment given may not be homogeneous, and indeed, in some cases, the parameter being addressed (mitotic score as a grading component) may have affected the original systemic treatment decision. This may limit the ability of the prognostic analyses in making assertions that either mitotic counts or Ki-67LI can identify a group in which chemotherapy could be withheld.

## Conclusion

In conclusion, proliferation assessment by using Ki-67LI and MS can distinguish subgroups of patients with luminal/HR^+ ^BC with significantly different clinical outcomes. Overall, both showed comparable results, with Ki-67LI having a marginal advantage in terms of patient-cohort separation. Neither MS nor Ki-67LI has additional prognostic value in HER2^+ ^and TNBC. This study emphasises the importance of determination of appropriate clinically relevant cut points for Ki-67LI and demonstrates that sufficient IHC assessment of Ki-67LI can be achieved by using a single FFS. This method could provide a cost-effective method for prognostic subclassification of luminal/HR^+ ^BC in routine clinical practice.

## Abbreviations

CISH: chromomeric *in situ *hybridization; ER: estrogen receptor; FFPE: formalin-fixed paraffin-embedded; HER2: human epidermal growth factor receptor 2; IHC: immunohistochemistry; Ki-67LI: Ki-67 labeling index; MSs: mitotic frequency scores; PR: progesterone receptor; TN: triple negative; TNnon-B: triple-negative non-basal.

## Competing interests

The authors declare that they have no competing interests.

## Authors' contributions

MA performed the practical work, performed the statistical analysis, and drafted the manuscript. EAR conceived and designed the project, participated in the statistics and in writing the manuscript. AAB, FB, and ARG participated in the practical work and contributed to the manuscript. IOE and JSR-F contributed to the project design and to the manuscript. All authors read and approved the final manuscript.
